# A bittersweet symphony: genetic insights into cider apple fruit quality

**DOI:** 10.1093/g3journal/jkaf241

**Published:** 2025-10-19

**Authors:** Pierre Bouillon, David Zakalik, Michael Brown, Shanthanu Krishna Kumar, Gregory Peck

**Affiliations:** School of Integrative Plant Science—Horticulture, Cornell University, 135 Plant Science Building, Ithaca, NY 14853, United States; School of Integrative Plant Science—Horticulture, Cornell University, 135 Plant Science Building, Ithaca, NY 14853, United States; School of Integrative Plant Science—Horticulture, Cornell University, 135 Plant Science Building, Ithaca, NY 14853, United States; Department of Plant Science, The Pennsylvania State University, University Park, PA 16802, United States; School of Integrative Plant Science—Horticulture, Cornell University, 135 Plant Science Building, Ithaca, NY 14853, United States

**Keywords:** genome-wide association study (GWAS), germplasm, malic acid, *Malus domestica*, marker-assisted breeding, polyphenol, sugar

## Abstract

Many traditional cider apples (*Malus domestica*) have unique chemotypic traits that impact the sensory profile and fermentation characteristics of the final product. In particular, cider apples may have greater polyphenol, organic acid, and sugar concentration than fresh-market apples. Despite historic importance and a growing market in many parts of the world, the genetic basis underlying cider apple fruit quality remains poorly understood. Therefore, few functional genetic markers have been successfully adapted for cider apple breeding. Using a genome-wide association study on 253 cider apple accessions from the USDA *Malus* collection, we identified 19 significant marker-trait associations for fruit quality traits. Notably, we identified a distinct marker on chromosome 15 that was strongly associated with total polyphenols, a key determinant of bitterness and astringency. A major association on chromosome 16, near the Ma1 locus, explained a substantial proportion of the phenotypic variance for titratable acidity and pH, confirming the importance of this region. Using these 2 loci, we were able to distinguish between cider apple groups, especially for bittersweet apples. A major locus on chromosome 1 was linked to the ratio of glucose and sucrose. This locus could be targeted to select genotypes with increased glucose content, which could improve fermentation kinetics. Overall, these results provide a robust genetic analysis focusing on quality traits in a cider-specific germplasm, laying the foundation for identifying apple cultivars with desirable attributes for cider production from germplasm collections and for making marker-assisted selections within breeding programs.

## Introduction

Traditional cider apples have been selected for fermentation and are typically classified by their sweetness, sharpness, bitterness, and astringency (Miles et al. 2020). These specific attributes have led to the development of different classification systems used to distinguish cider apples (Karl et al. 2022). The most widely recognized system is the Long Ashton Research Station (LARS) classification system, which categorizes apples into 4 groups based on their tannin concentration and titratable acidity (TA): sharp (low tannin, high acid), sweet (low tannin, low acid), bittersweet (high tannin, low acid), and bittersharp (high tannin, high acid) (Brooke-Hunt 1904). The classification of cider apples is crucial for cider production, as the balance of sugars, acidity, and tannins directly impacts the sensory profile and fermentation characteristics of the final product (Lea and Drilleau 2003; Picchi et al. 2023). While some producers make ciders from a single cultivar, a balanced product is typically achieved by blending different apple cultivars to reach a targeted flavor profile (Lea and Drilleau 2003).

Acidity plays an important role in cider by affecting the perceived flavor (Karl et al. 2022), and during fermentation, high acidity levels (low pH) impact beverage stability by preventing microbial contamination (Karl et al. 2022). The predominant organic acid in apple fruit is malic acid, which accounts for more than 80% of the total acid content (Liu et al. 2022). Two major quantitative trait loci (QTLs) have been identified for acidity-related traits on chromosome 8 (Ma3 locus) and 16 (Ma1 locus) (Verma et al. 2019). These 2 loci have been successfully applied to classify cider apple germplasm for fruit acidity (Kumar et al. 2021), which confirm their potential utility for cider-specific apple breeding programs.

A distinguishing feature of many traditional French and English cider apples is the relatively high concentration of polyphenols (Lea and & Drilleau 2003). Polyphenols in apple fruit include hydroxycinnamic acids, flavan-3-ols, flavonols, anthocyanins, and dihydrochalcones (Chagné et al. 2012). Among them, flavan-3-ols strongly contributes to the astringency, bitterness, and mouthfeel of cider (Verdu et al. 2014). Significant progress has been achieved in understanding the genetic basis of phenolic content in apple fruit through linkage (Chagné et al. 2012; Verdu et al. 2014; Bouillon et al. 2024) and association analyses (McClure et al. 2019; Kumar et al. 2022; Li et al. 2025). Additional studies applied to cider apples have successfully detected loci associated with phenolic content (Verdu et al. 2014; Leforestier et al. 2015). However, identified regions are currently too numerous and/or too large to be easily applied in marker-assisted breeding (Miles et al. 2020).

Among the many QTLs detected, numerous overlaps have been reported at the top of chromosome 16 for both cider (Verdu et al. 2014; Watts et al. 2023) and fresh-market apples (Chagné et al. 2012; McClure et al. 2019; Bouillon et al. 2024). A candidate gene encoding a leucoanthocyanidin reductase, *LAR1*, has been linked to this metabolite quantitative trait loci (mQTL) hotspot (Khan et al. 2012). Notably, Ma1 locus and LAR1 locus are close located on top of chromosome 16 (Liao et al. 2021). Recent genetic evidence (Liao et al. 2021) suggests that this genetic region may have undergone 2 different selection processes between cider and fresh-market apples. Signatures of selective sweeps at the Ma1 locus, rather than LAR1, have been identified in fresh-market apples, indicating that selection for fruit acidity has played a key role in shaping fruit taste during apple domestication and breeding (Liao et al. 2021). In contrast, a signature of selection at the major LAR1 locus, rather than Ma1, was detected in cider apples, suggesting the importance of flavan-3-ols contents in cider apples (Liao et al. 2021). However, previous studies were either limited to biparental populations (Verdu et al. 2014) or included cider apples only as a minor subset of genome-wide association study (GWAS) germplasms (Watts et al. 2023; Liao et al. 2021). As a result, they may not fully capture the genetic diversity present within cider apples, limiting our understanding of loci associated with traits unique to these use specific apples. Further studies are currently needed to better understand the genomic differences that could lead to phenotypic differentiation among cider and fresh-market apples.

During fermentation, yeast metabolizes the soluble sugars, fructose, sucrose, and glucose into ethanol. Thus, the soluble solids concentration (SSC), a reference index that is typically used to quantify soluble sugars, will directly influence alcohol by volume concentration in the final product. Sorbitol, a sugar alcohol, is nonpreferentially fermented by yeast and will leave a residual sweetness that can be a positive sensory quality in cider (Miles et al. 2020; Karl et al. 2022). Among the sugars, fructose is found in the greatest concentration in apples (Karl et al. 2022). Sugar production, accumulation, transportation, and utilization are complex traits related to all plant metabolic activity. QTLs related to sugar content have been detected on all 17 apple chromosomes (Yuan et al. 2023), but 2 main clusters have been successfully linked to regulatory genes for sugar content on LG01 (fructose and sucrose) and LG03 (glucose) offering potential breeding applications (Liao et al. 2021; Zhang et al. 2024). Another locus on LG10 has been linked to sorbitol accumulation (Liao et al. 2021).

Although some cider-specific breeding has occurred in the United States (United States Department of Agriculture, Washington State University), England (Long Ashton Research Station), France (Institut Français des Produits Cidricoles), and Spain (Instituto Técnico de Gestión Agrícola), there have been few commercial releases in the last several decades. Indeed, apple breeding efforts have primarily focused on fresh-market cultivars, with less emphasis on cider cultivars. While some breeding objectives are concomitant with cider apple improvement, these programs can select against beneficial traits for cider production, such as high tannin and high acid content (Miles et al. 2020). Despite their practical importance, the genetic basis underlying the variation in cider apple fruit quality traits remains poorly understood (Miles et al. 2020). Past genetic studies have been mainly applied to fresh-market apple families/germplasm, and few functional genetic markers have been successfully adapted for cider selection (Miles et al. 2020).

In this study, we performed a GWAS on an extensive cider germplasm (*N* = 253) from the USDA *Malus* collection (Volk et al. 2022) with the goal of establishing the genetic basis of key fruit quality traits in cider apples for future application in cider-specific apple breeding program.

## Materials and methods

### Plant material and phenotyping

A subset from the National Plant Germplasm System (NPGS) *Malus* germplasm collection—maintained by the US Department of Agriculture (USDA) Plant Genetics Resource Unit (PGRU) in Geneva, NY (lat. 42°53′40.3″N, long. 77°00′23.8″W), were selected based on their historic, current, and potential use for cider production (Kumar et al. 2021). After removing accessions that were triploids and duplicates, a total of 253 diploid genotypes were used for this study.

Apples were harvested at maturity (Starch Pattern Index > 6 on the Blanpied and Silsby 8-point scale) and stored at 4 °C under ambient atmospheric gases for 1 to 4 wk before fruit processing analysis at Cornell University in Ithaca, NY. A total of 15 fruits per genotype were sampled and randomly divided into 3 batches of 5 fruits. Each batch was milled and pressed in a juicer (Model 280; Norwalk Juicers, Bentonville, AR). Juice from each replicate was then stirred and aliquoted into 50-mL tubes. Juice samples were stored at −80 °C until analysis (Kumar et al. 2021).

Samples were thawed to room temperature and vortexed for 10 s. Juice TA and pH were determined using an automatic titrator (Unitrode pH meter, 778 sample processor, and 800 Dosino dosing device; Metrohm, Herisau, Switzerland). TA was assessed by titrating a 5-mL juice aliquot with a standardized 0.1-N NaOH solution (VWR Chemicals, Radnor, PA, USA) to a pH endpoint of 8.1 and expressed in grams per liter of malic acid equivalents (g/L MAE) using the following formula: TA (g/L MAE) = (mL NaOH to endpoint × 67)/(mL juice sample), where 67 is the malic acid equivalence factor.

Total polyphenol concentration was determined using the Folin–Ciocalteu assay with an 8-point standard calibration curve ranging from 0 to 3.0 g L⁻¹ of gallic acid. The reaction was conducted in a CELLSTAR 96-well microplate (Greiner Bio-One, Monroe, NC, USA). Each reaction mixture contained 1.5 µL of standard or sample, 34.9 µL of water, and 90.9 µL of Folin–Ciocalteu reagent (Sigma-Aldrich, St. Louis, MO, USA). After a 6-min incubation, 72.7 µL of 7% (w/v) sodium carbonate solution (Sigma-Aldrich) was added, and the mixture was incubated at 21 °C in the dark for 1 h. Absorbance was measured at 765 nm using a SpectraMax 384 Plus microplate reader (Molecular Devices, San Jose, CA, USA). Total polyphenol content was calculated by linear regression from the standard curve and adjusted for dilution when necessary. Results were expressed as gallic acid equivalents (g/L GAE) in apple juice samples.

Individual sugar contents were determined by high-performance anion exchange chromatography with pulsed amperometric detection (HPAE-PAD) as described in Kamenetsky et al. (2003). Samples were analyzed on a DIONEX DC ICS-5000 series chromatograph, equipped with a CarboPac PA-1 column, a pulsed amperometric detector, and a gold electrode (Thermo Fisher, Pittsburgh, PA, USA). Elution was performed at a flow of 1.0 mL/min at about 1350 psi with 200-mM NaOH (Fisher Scientific, Pittsburgh, PA, USA) during a 20-min run. The amounts of sorbitol, glucose, fructose, and sucrose were then quantified by comparison with a 7-point calibration curve generated from known standards (Sigma-Aldrich, St. Louis, MO, USA).

### Genotyping data

Genotyping data were obtained from a 20-K SNP array (Bianco et al. 2014) as part of an ongoing collaborative apple pedigree reconstruction project (Muranty et al. 2020; Luby et al. 2022; Durel et al. 2023). The data were curated following the methods described by Vanderzande et al. (2019), and a subset of 10,321 SNP markers considered robust were used for subsequent genetic analyses (Supplementary Table 2) (Howard et al. 2021).

### Statistical analysis

Adjusted phenotypic mean of each genotype across years (2017–2021) was estimated using a linear model with genotype and year as fixed effects (Jung et al. 2020) as the following:


$$P_{ik}=\mu +g_{i}+Y_{k}+e_{ik}$$

where $P_{ik}$ is the phenotypic value of the *i*th genotype on the *k*th year, *μ* is the grand mean, $g_{i}$ is the effect of the *i*th genotype, $Y_{k}\;$ is the effect of the *k*th year on the trait; and $e_{ik}$ is the residual term of the model.

Broad-sense heritability ($h^{2}$) was estimated as:


$$h^{2}=\;\frac{\sigma_{G}^{2}}{\sigma_{G}^{2}+\left(\frac{\sigma_{\varepsilon }^{2}}{n_{r}}\right)}$$

where $\sigma_{G}^{2}$ is the variance of genotype effect, $\sigma_{\varepsilon }^{2}$ is the variance of residual term, and $n_{r}$ is the average number of replicates over both years.

Least square means (LS-means) of traits for each genotype were extracted using the R package “lsmeans” (Lenth 2016) and used later for GWAS. Values greater than 3 standard deviations from the mean were considered outliers and subsequently removed from further analysis to avoid detection of artifact loci (Alvarez Prado et al. 2019). To assess between-trait relationships, *x*–*y* plots and correlation coefficients were analyzed (Supplementary Fig. 1). Principal component analysis (PCA) was performed on phenotypic data (Supplementary Fig. 3).

### Genome-wide association study

GWAS was conducted using the R package GAPIT 3.0 (Wang and Zhang 2021), which implements the Bayesian-information and linkage-disequilibrium iteratively nested keyway (BLINK) model (Huang et al. 2019). This model is currently recognized as one of the most powerful methods in terms of both computational efficiency and statistical power (Huang et al. 2019; Wang and Zhang 2021; Yoosefzadeh-Najafabadi et al. 2022) and was therefore selected to perform analyses. GWAS was performed on an *n* × *m* matrix with a population size of *n* = 253 genotype and *m* = 9,566 markers. SNPs were filtered for minor allele frequency (MAF) < 0.05. The first 5 principal components (Supplementary Fig. 6; Supplementary Table 3) were fitted as covariate variables to reduce false positives due to population stratification. The kinship matrix (Supplementary Fig. 7; Supplementary Table 4) was computed using the VanRaden algorithm (VanRaden 2008). Marker-trait associations (Supplementary Table 5) were considered significant using a Bonferroni-corrected threshold α = 0.05, corresponding to −log_10_(p) > 5.28.

To assess differences in phenotypic values across the most significant SNP [phenotype variance explained {PVE} > 20%], a Kruskal–Wallis test was conducted using the ggbetweenstats function from the R package (Patil 2021). Post hoc pairwise comparisons were conducted using Dunn's test, with Holm's adjustment applied to control for multiple comparisons. The gradual evidence language was used to report statistical results (Muff et al. 2022). To investigate candidate genes at proximity of most significant SNPs, candidate genes were screened within a 3-Mb window using the functional annotation of the “GGDH13.v1.1” apple genome (Daccord et al. 2017) as a reference. All statistical analysis and data formatting were performed with R version 4.4.1.

## Results

### Variations and relationship between traits

Phenotypic values for total polyphenols (TP), TA, pH, SSC, and individual sugars (glucose, fructose, sucrose, and sorbitol) are reported in Supplementary Table 1. Between-trait correlations were estimated with Pearson correlation coefficient (Supplementary Fig. 1). TA and pH were highly correlated (*r* = −0.87). TP and pH exhibited positive correlation with *r* = 0.36. Individual sugar concentrations were also correlated with SSC that exhibited *r* of 0.58, 0.53, 0.35, and 028, for sorbitol, sucrose, fructose, and glucose, respectively. Fructose was correlated to glucose (*r* = 0.61). Sorbitol concentration was correlated to pH, glucose, and fructose (*r* of 0.42, 0.39, and 0.36, respectively). Fructose and sucrose amounts were not correlated. To further assess relationships among traits, PCA was conducted (Supplementary Fig. 2). The 4 first principal components (PCs) accounted for 37.6%, 17.5%, 15%, and 13.9% of the total variance, respectively. TA and pH were negatively correlated on dimension 1 vs dimension 2. Individual sugar amounts were also correlated on dimension 1 vs dimension 2.

Least-square phenotypic means exhibited substantial variation among cultivars (Supplementary Fig. 3). Phenotypic means for TP varied from −0.41 to 3.36. SSC ranged from 8.40 to 15.69. Acidity-related traits, including TA and pH values, ranged from 0.21 to 14.46 (g/L GAE) and 2.90 to 5.30, respectively. Phenotypic means for TA and pH exhibited a bimodal distribution, as previously reported for this population (Kumar et al. 2021). Phenotypic means for individual sugar amounts also showed important variation across cultivars, with glucose ranging from −0.41 to 49.05, fructose from 25.41 to 116.38, sucrose from −1.70 to 101.01, and sorbitol from −5.26 to 28.20. The proportion of fructose among individual sugars ranged from 25.1% to 73.9%.

### Marker-trait associations

**Fig. 1.**
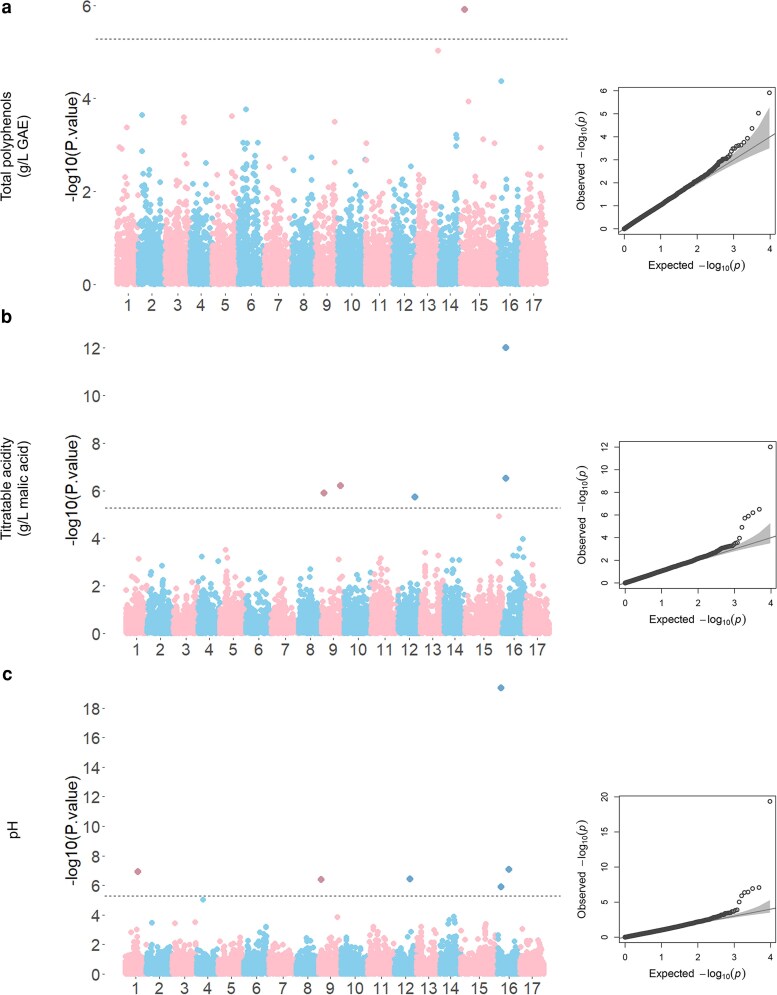
Manhattan and QQ plots for GWAS of titratable acidity (TA), total polyphenols (TP), and pH. Manhattan plots (left) display the genome-wide association results for a) TA, b) TP, and c) pH. The *x* axis represents the 17 chromosomes, and the *y* axis shows the −log10(P-value) for each SNP. The dashed horizontal line represents the Bonferroni-corrected significant threshold. Alternating colors distinguish different chromosomes. The QQ plots (right) compare the observed vs expected −log10(P) distributions.

Significant marker-trait associations were detected for: TP, TA, pH, glucose, and sucrose (Figs. 1 and 3a). A total of 19 marker-trait associations were detected. Information about markers associated with traits: SNP index, SNP name, chromosome and position in megabase-pair (Mb), *P*-value, minor allele frequency (MAF), effect size, and PVE are reported in Table 1. Manhattan plots for SSC, fructose, and sorbitol can be found in the Supplementary material (Supplementary Fig. 4).

**Table 1.** Summary of significant SNP–trait associations for titratable acidity (TA), pH, total polyphenols (TP), glucose, and sucrose.

Trait	h^2^	SNP index	Chromosome	Position (cM)	Position (Mb)	*P*-value	MAF	Effect Size	PVE (%)
TA	0.936	13732	9	7.21	2.15	1.26E−06	0.18	−1.13	8.64
TA	0.936	14306	9	55.27	29.92	6.30E−07	0.06	−1.68	10.19
TA	0.936	14851	12	48.46	27.90	1.91E−06	0.17	−0.99	8.63
TA	0.936	7966	16	9.06	3.16	9.78E−13	0.43	−1.93	38.89
TA	0.936	1542	16	10.43	3.41	3.12E−07	0.34	−1.50	6.33
pH	0.943	9232	1	37.7	25.14	1.16E−07	0.39	−0.12	0.64
pH	0.943	13732	9	7.21	2.15	4.27E−07	0.18	0.17	5.59
pH	0.943	14837	12	46.17	27.09	3.68E−07	0.31	0.13	1.93
pH	0.943	7966	16	9.06	3.16	4.36E−20	0.43	0.38	67.43
pH	0.943	1542	16	10.43	3.41	1.24E−06	0.34	0.20	2.74
pH	0.943	8055	16	32.86	8.48	8.37E−08	0.22	−0.16	4.17
TP	0.772	7039	15	11.51	3.21	1.22E−06	0.14	−0.36	45.89
Glucose	0.765	9350	1	48.54	28.38	2.04E−15	0.46	−5.30	20.07
Glucose	0.765	13633	8	60.66	28.29	6.92E−08	0.28	3.24	7.72
Glucose	0.765	1691	10	58.3	33.84	1.84E−07	0.21	3.30	6.47
Glucose	0.765	1542	16	10.43	3.41	3.60E−07	0.34	3.81	15.52
Sucrose	0.874	9347	1	48.54	28.38	2.17E−09	0.43	7.51	23.01
Sucrose	0.874	2907	6	54.79	32.10	2.16E−08	0.09	10.30	13.48
Sucrose	0.874	3445	10	6.49	3.68	3.63E−09	0.17	−8.87	21.35

For each SNP, the table reports broad-sense heritability values for the phenotypic trait (h^2^), SNP index, chromosome, position (in cM and Mb), *P*-value, minor allele frequency (MAF), effect size, and the percentage of phenotype variance (PVE) explained by the SNP.

A marker-trait association was observed for TP on chromosome 15 (SNP 7039) (Fig. 1; Table 1), which explained 45.89% of the phenotypic variations. This hit was located 2 to 3-Mb upstream 2 candidate genes: *PH4* (MD15G1051000) and *MdMYB9* (MD15G1051400). For TA, 5 marker-trait associations were detected on chromosome 9 (SNPs 13732 and 14306), chromosome 12 (SNP 14851), and chromosome 16 (SNPs 7966 and 1542). For TA (Fig. 1; Table 1), 5 marker-trait associations were detected on chromosomes 9, 12, and 16. Two markers (SNPs 13732 and 14306) were detected on chromosome 9 at 2.15 and 29.92 Mb, explaining respectively 8.64% and 10.19% of the phenotypic variance. One marker (SNP 14851) was detected on chromosome 12 at 27.90 Mb with a PVE of 8.63%. A GWAS hit was detected on chromosome 16 at 3 Mb. The most significant SNP (SNP 7966) explained 38.89% of the phenotypic variance. This SNP was located at 3.16 Mb, 200 kb upstream Ma locus. Excepting SNP 14306, all these markers were also detected for pH (Fig. 1; Table 1). Another locus was found for pH on chromosome 1 (SNP 9232—25.14 Mb) with a PVE of 0.64%.

Among sugar-related traits, 7 significant marker-trait associations were detected for glucose and sucrose amounts (Fig. 3a; Table 1). A shared marker-trait association was detected on chromosome 1 at 28.38 Mb (SNPs 9347 and 9350) for glucose and sucrose, which explained 20.07% and 23.01% of the phenotypic variance, respectively. This locus was located 2 Mb upstream *MdRPM1*, a candidate gene for sucrose content. As the 2 SNPs were located next from each other, only SNP 9347 was kept for further analysis. Three other SNPs (SNPs 13633, 1691, and 1542) were detected for glucose content on chromosomes 8, 10, and 16 with PVEs of 7.72%, 6.47%, and 15.52%, respectively. Two candidate genes were located upstream (<1000 kb) the detected locus on chromosome 8: a sucrose transport gene *MdSUT3* (MD08G1209900) and a neutral invertase *MdNINV7* (MD08G1217200). Two other SNPs (SNPs 2907 and 3445) were detected for sucrose content on chromosomes 6 and 10 with PVEs of 13.48% and 21.35%, respectively. A candidate gene encoded 1 enolase *MdENO1* (MD06G1208300), was located 2,000 kb downstream the detected marker-trait association on chromosome 6. A sucrose-phosphate synthase *MdSPS4* (MD10G1002500) was located 3 Mb upstream the detected marker-trait association on chromosome 10.

### Identification of important alleles for cider-specific traits

**Fig. 2.**
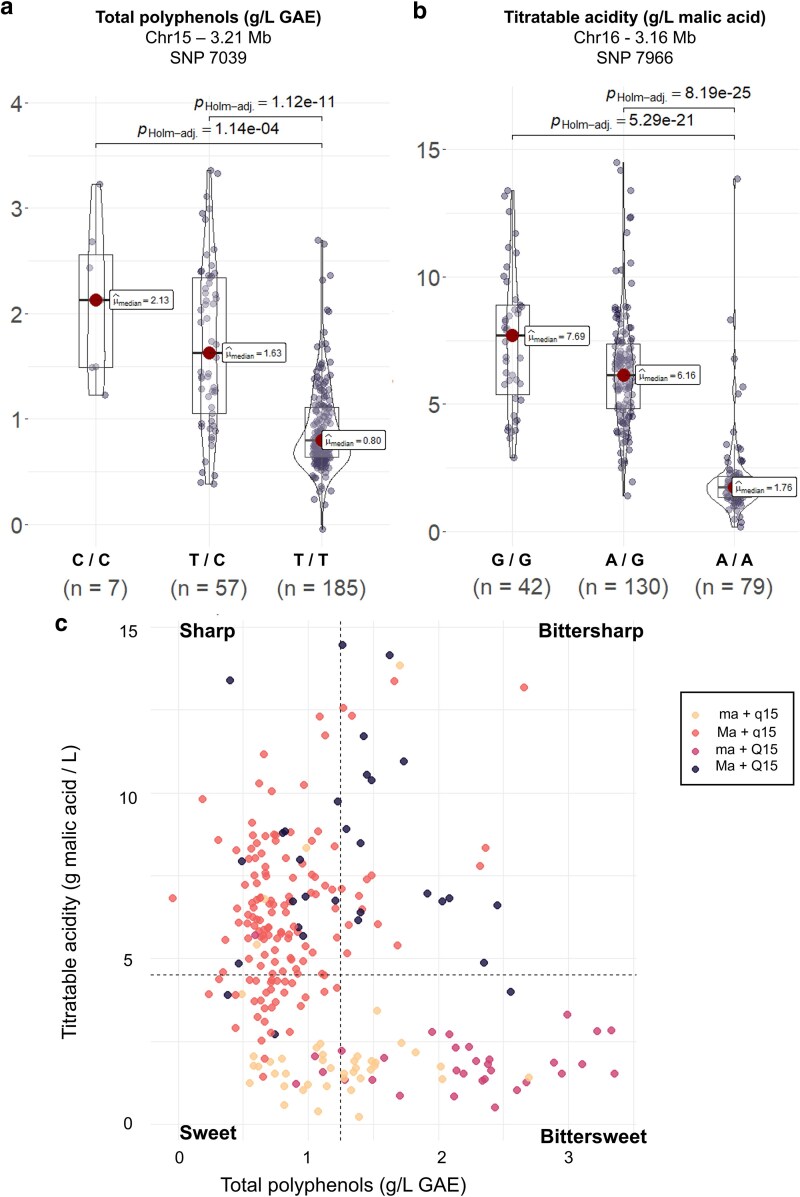
Genotypic effects of significant SNPs and their relationship with total polyphenols (TP) and titratable acidity (TA). Genotypic effects of SNP 7039 (Chr15, 3.21 Mb) on TP (a) and SNP 7966 (Chr16, 3.16 Mb) on TA (b). To assess differences in phenotypic values among genotypes, a Kruskal–Wallis test was conducted. Post hoc pairwise comparisons were conducted using Dunn's test, with Holm's adjustment applied to control for multiple comparisons. Each box plot represents the distribution of trait values across different genotypic groups, with median values highlighted. The statistical evidence for differences between genotypes (p Holm-adj) are displayed. c) Scatter plot depicting the relationship between TP and TA, with points colored by allelic combinations at the significant loci (Ma and Q15). For SNP 7039 (Q15 locus), C/C and T/C genotypes were denoted Q15, and T/T genotypes were denoted q15. For SNP 7966 (Ma1 locus), G/G and G/A genotypes were denoted Ma1, and A/A genotypes were denoted ma. LARS classification threshold were highlighted by dashed lines: sweet [TP < 1.25 g/L gallic acid equivalents (GAE) and TA < 4.5 g/L malic acid equivalents (MAE)], bittersweet (TP > 1.25 g/L GAE and TA < 4.5 g/L MAE), sharp (TP < 1.25 g/L GAE and TA > 4.5 g/L MAE), and bittersharp (TP > 1.25 g/L GAE and TA > 4.5 g/L MAE).

**Fig. 3.**
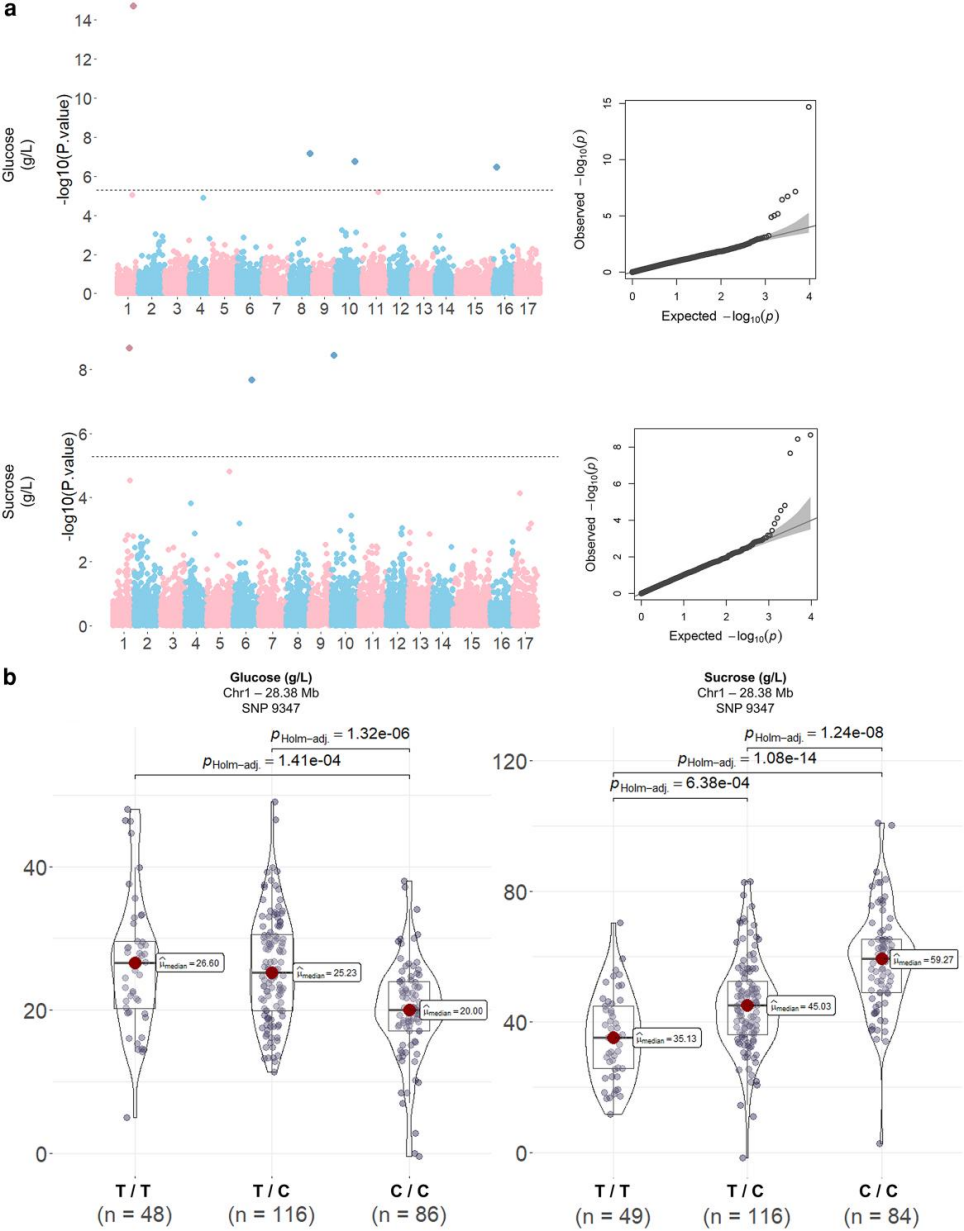
GWAS and genotype–phenotype associations for glucose and sucrose content. a) Manhattan plots for glucose and sucrose content. The *x* axis represents the 17 chromosomes, and the *y* axis shows the −log10(*P*-value) for each SNP. The dashed horizontal line represents the Bonferroni-corrected significant threshold. Alternating colors distinguish different chromosomes. The QQ plots (right) compare the observed vs expected −log10(P) distributions. b) Genotypic effects of SNP 9347 (Chr1, 28.38 Mb) on glucose (left) and sucrose (right) content. To assess differences in phenotypic values among genotypes, a Kruskal–Wallis test was conducted. Post hoc pairwise comparisons were conducted using Dunn's test, with Holm's adjustment applied to control for multiple comparisons. Violin plots show the distribution of phenotypic values across the 3 genotypic groups (T/T, T/C, and C/C), with median values indicated. Pairwise comparisons were conducted using Holm-adjusted *P*-values.

The most significant marker-trait associations were further investigated to identify important alleles for cider-specific traits (Figs. 2a and b and 3b). Comparison among genotypes for SNP 7039 revealed very strong evidence that this locus was associated with TP contents (Fig. 2a). C/C and T/C genotypes had mean values of 2.10 and 1.71, respectively, 2 times greater than T/T genotypes, which had mean values of 0.92. Differences between C/C and T/C genotypes could not be hypothesized due to limited sample size (*n* = 7 for C/C genotypes). Considering acidity-related traits, comparison among genotypes for SNP 7966 revealed very strong evidence that this locus was associated with differences in TA (Fig. 2b) and pH (Supplementary Fig. 5) values. G/G and G/A genotypes exhibited higher TA values, and lower pH values, in comparison to A/A genotypes. G/G and G/A genotypes showed mean values of 7.49 and 6.33, respectively, more than 3 times greater than A/A genotypes, which had a mean value of 2.16. A similar trend was observed for pH, with G/G and G/A genotypes showing lower pH values (mean values of 3.54 and 3.41, respectively) in comparison to A/A genotypes (mean value of 4.43). Comparison among allelic combinations suggested that “G” allele was dominant on “A” allele (there was no statistical difference between homozygotes and heterozygotes). Classification (Fig. 2c) based on these 2 loci was achieved based on allelic combinations. For SNP 7039, C/C and T/C genotypes were denoted Q15 because they owned at least 1 favorable allele for TP, and T/T genotypes were denoted q15. For SNP 7966, G/G and G/A genotypes were denoted Ma, and A/A genotypes were denoted ma. Genotypes were plotted for TP and TA to confirm overlap between traditional classification system and classification based on these 2 loci. Proportions of well-classified genotypes based on SNPs 7966 and 7933 were estimated for each class (Table 2).

**Table 2.** Classification of apple genotypes based on total polyphenols (TP) and titratable acidity (TA) grouped by SNPs 7966 and 7939.

	Bittersharp	Bittersweet	Sharp	Sweet	Well-classified (%)
**ma + q15**	1	19	3	20	46.51
**Ma + q15**	15	0	96	31	67.61
**ma + Q15**	0	28	1	3	87.50
**Ma + Q15**	15	1	12	2	50.00
				**Overall**	62.91

The table presents the count of genotypes in each LARS class (bittersharp, bittersweet, sharp, sweet) along with the percentage of well-classified genotypes for each marker. LARS classification was used to grouped genotypes as sweet (TP < 1.25 g/L GAE and TA < 4.5 g/L MAE), bittersweet (TP > 1.25 g/L GAE and TA < 4.5 g/L MAE), sharp (TP < 1.25 g/L GAE and TA > 4.5 g/L MAE), and bittersharp (TP > 1.25 g/L GAE and TA > 4.5 g/L MAE).

Comparison among genotypes for SNP 9347 confirmed very strong evidence that this locus was associated with glucose and sucrose contents (Fig. 3b). T/T and T/C genotypes (mean values of 26.60 and 25.23, respectively) exhibited 1.25 times higher glucose contents than C/C genotypes (mean value of 20.00). In contrast, C/C genotypes (mean value of 59.27) exhibited from 1.25 to 1.6 times higher sucrose contents than T/T and T/C genotypes (mean values of 35.13 and 45.03). There was also strong evidence that T/C genotypes displayed greater sucrose content than T/T.

## Discussion

### Identification of SNPs associated with cider apple quality traits

Marker-trait associations were detected for TP, TA, pH, glucose, and sucrose (Figs. 1 and 3a), while no significant association was found for SSC, fructose, or sorbitol. Among the 19 marker-trait associations detected, 3 loci—LG16 for TA and pH, LG15 for TP, and LG1 for sucrose content—have been reported in previous studies (Watts et al. 2023; Liao et al. 2021; Larsen et al. 2019). Another 11 marker-trait associations are previously unreported. Colocalization between loci identified for pH and TA, and for glucose and fructose, confirmed that these traits were interconnected.

We were able to identify associations for sucrose and glucose, but no clear association was detected for SSC, fructose, or sorbitol (Fig. 3a; Supplementary Fig. 4). The complex regulation of sugar metabolism is controlled by many genetic, physiological, and environmental factors (Yuan et al. 2023). It is also possible that the absence of strong genetic associations indicates the absence of larger effect alleles segregating in our germplasm (Clauw et al. 2025).

### Candidate genes identified through GWAS contribute to important phenotypic variations for cider production

Tannin content is one of the most distinctive traits between cider and fresh-market apples (Leforestier et al. 2015). Previous GWAS studies identified two strong marker-trait associations for TP on chromosomes 15 and 16 (Lin et al. 2023; Watts et al. 2023). The locus reported on chromosome 15 colocalized with our findings confirming the association between a region around 3 Mb on chromosome 15 and phenolic contents in cider apples. Two candidate genes that encoded MYBs, *PH4* (MD15G1051000) and *MdMYB9* (MD15G1051400), could be involved in tannin biosynthesis. *MdMYB9* regulates both anthocyanin and procyanidin synthesis (An et al. 2015), while *PH4* is mostly involved in vacuolar acidification (Lin et al. 2023). This genetic region now needs to be narrowed using a higher marker density. While this study relied on a limited set of SNP markers from the 20K SNP array (Bianco et al. 2014), our marker density corresponds to approximately 1 marker every ∼68 kb, which may limit the detection of associations. Future projects will focus on obtaining higher-resolution marker data.

We did not detect associations on chromosome 16 for TP. In our study, TP were estimated using the Folin–Ciocalteu assay, which lacks the specificity to differentiate among classes of phenolic compounds and instead provides a single overall measurement of phenolic compounds (Tyagi et al. 2025). Indeed, the proportion of flavan-3-ols among TP can vary greatly depending on the genotype (Tyagi et al. 2025), which is a limitation for the detection of a genetic association. Another hypothesis is that differences in genotypes used in the study could explain the absence of clear segregation for this locus. Watts et al. (2023) used a germplasm consisting of cider and fresh-market apples, while in our study, we focused on apple cultivars primarily used for cider production. Future studies should identify the genetic determinism of individual phenolic compound accumulation in cider apples.

While TA is a major quality trait involved in acidity/sourness perception, pH is a key phenotypic trait for cider making, with an ideal pH range (3.2 to 3.8) avoiding cider spoilage (Kumar et al. 2021). Two major QTLs, Ma and Ma3, are involved in regulation of apple fruit acidity (Verma et al. 2019). Further research (Bai et al. 2012) identified 2 aluminum-activated malate transporter-like genes, *MdMa1* and *MdMa2*, locating at the Ma locus. A natural mutation in the Ma1 gene, leading to a truncated protein that reduces malate transport and is associated with lower fruit acidity, has been identified in apple (Bai et al. 2012). A previous study reported that Ma1 could be used to predict acidity in cider apples (Kumar et al. 2021). Our results confirmed that Ma1 locus is the main genetic factor regulating fruit acidity in cider apples with PVEs of 38.89% and 67.43% for TA and pH, respectively (Table 1). Comparison of TA and pH values across SNP 7966 genotypes (Fig. 2b) confirmed the dominant effect of “Ma” allele over the recessive “ma” allele. However, we observed important phenotypic variations among heterozygous (from 1.41 to 14.5 g/L MAE content and from 3.08 to 4.96 for pH values) confirming that other loci are likely involved in fruit acidity metabolism. Indeed, two marker-trait associations on chromosome 9 (2 Mb) and 12 (27 Mb) were detected for pH and TA with cumulative PVEs of 18.82% and 7.52%, respectively. Further studies should develop combined functional markers for fruit acidity that would be applicable in cider apple breeding programs.

Our study identified significant marker-trait associations on chromosome 1 for glucose and sucrose contents (Fig. 3b) that colocalized with a previously described cluster in fresh-market apples (Larsen et al. 2019; Liao et al. 2021; 20). This cluster has been associated with both fructose and sucrose contents (Guan et al. 2015; Larsen et al. 2019; Liao et al. 2021). A candidate gene for fruit sucrose content, MdRPM1-like (MD01G1186600), was located 1,500 kb downstream marker-trait association (SNP 9347). Liao et al. (2021) showed that expression of MMdRPM1-like was correlated with sucrose accumulation, but not with glucose accumulation (Liao et al. 2021). In our study, we observed that the ratio of sucrose to glucose accumulation depends on genotypes at this locus (Fig. 3b). Comparison of sucrose and glucose contents across SNP 9347 genotypes revealed very strong evidence that T/T and T/C genotypes exhibited higher glucose content and lower sucrose content than C/C genotypes. Allelic variations at this locus could be associated with differential accumulation of sucrose and glucose. Two candidate genes that could affect glucose content were identified on chromosome 8: a sucrose transporter, *MdSUt3* (MD08G1209900), which plays a key role in sucrose unloading (Li et al. 2012), and a neutral invertase, *MdNINV7* (MD08G1217200), which might be involved in sucrose decomposition into glucose and fructose (Zhang et al. 2024). Candidate genes for sucrose content were identified on chromosomes 6 and 10: a sucrose phosphate synthase, *MdSPS4* (MD10G1002500), which was correlated with sucrose accumulation in apple (Li et al. 2012), and Enolase 1 *MdENO1* (MD06G1208300) that has been associated with sugar metabolism (Cao et al. 2024).

### Genetic basis of cider apple classification: a breeding perspective

Cider apple classification is traditionally based on polyphenol concentration and acidity. Our results indicated that these 2 traits are primarily regulated by two loci on chromosomes 15 and 16 (Fig. 2a and b). Using these loci, we were able to classify our germplasm accordingly (Fig. 2c). This classification coincided with the four recognized classes of cider apples (Karl et al. 2022). Cultivars within the respective genotype groups—ma + q15 (orange), Ma + q15 (red), ma + Q15 (pink), and Ma + Q15 (blue)—were predominantly distributed into the “sweet,” “sharp,” “bittersweet,” and “bittersharp” classes, respectively, with percentages of well-classified genotypes of 46.51%, 67.61%, 87.50%, and 50.00%, respectively (Table 2). The overall percentage of well-classified genotypes was 62.61%, indicating that the combination of these two loci can be efficiently used in cider breeding programs. Moreover, the bittersweet class achieved the highest proportion of well-classified genotypes (87.50%). Given the importance of bittersweet apples in cider making (Merwin et al. 2007), these results will help in selecting apple cultivars suitable for cider production. Interestingly, bittersharp apples showed the lowest percentage of well-classified genotypes (50.00%), suggesting that, despite the simple determinism of malic acid content in apple (linked to the presence of the Ma locus), other genetic factors are involved in the genetic control of high-acid and high-bitter accessions.

Sweet and sharp apples, commonly used in cider making, are generally culinary cultivars, whereas bittersharp and bittersweet apples are less commonly available (VanderWeide et al. 2022; Karl et al. 2022). Using these two loci, breeders can select for cider apples that belong to a specific category, suitable for producing particular styles of cider. Given the strong influence of crop load, season, and environment on apple classification (VanderWeide et al. 2022; Karl et al. 2022), future studies should also consider the genotype × environment × management interactions to aid in selecting ideotypes that are well suited for specific growing conditions.

Sugar content is a key factor in cider production, directly affecting potential alcohol concentration and residual sweetness (Karl et al. 2022). We did not identify marker-trait associations for this predominant sugar despite important variations in our germplasm population for fructose content (from 25.41 to 116.38 g/L). Limitations in detecting associations may be due to the complex regulation of sugar concentration in apple fruit, which is dependent on total synthesis, starch hydrolysis, source–sink relationships (i.e., fruit loading), environmental factors, and management (Yuan et al. 2023). Further studies should be addressed to better understand the interactions between genetic, environmental, and management factors, such as crop load, that affect sugar concentration for apple fruits.

Our results showed a 3-fold variation in the proportion of fructose relative to the other sugars (from 25.1% to 75.9%) in the evaluated cider germplasm. During fermentation, yeast metabolizes fructose, sucrose, and glucose, with a preference for glucose over the other sugars (Ferremi Leali et al 2024). Our study identified an association on chromosome 1 that was linked to the ratio of glucose/sucrose. Allelic variations associated with glucose content could be targeted to develop functional markers for selecting genotypes with higher glucose content, potentially enhancing fermentation capacity.

The cider industry is one of the fastest-growing sectors in the craft beverage market, creating new marketing opportunities for apple growers (Miles et al. 2020). However, the limited history and experience in selecting, cultivating, and utilizing cider apple cultivars present a significant challenge to this growing market (Miles et al. 2020). In this study, we provide the foundation for the future development of functional markers applicable in cider-specific apple breeding programs. These results would help breeders to generate new apple cultivars suitable for high-quality cider production.

## Supplementary Material

jkaf241_Supplementary_Data

## Data Availability

The data underlying this article are available in the article and in the Supplementary material. Supplemental material available at *G3* online.
