# Correction to: A bittersweet symphony: genetic insights into cider apple fruit quality

**DOI:** 10.1093/g3journal/jkag103

**Published:** 2026-06-18

This is a correction to: Pierre Bouillon, David Zakalik, Michael Brown, Shanthanu Krishna Kumar, Gregory Peck, A bittersweet symphony: genetic insights into cider apple fruit quality, *G3 Genes|Genomes|Genetics*, Volume 16, Issue 1, January 2026, jkaf241, https://doi.org/10.1093/g3journal/jkaf241

In the ‘Materials and Methods’ section, under the ‘Genome-wide association study’ heading, a statement has been corrected to read:

“Marker–trait associations (Supplementary Table 5) were considered significant using a Bonferroni-corrected threshold α = 0.05, corresponding to −log10(p) > 5.28.” instead of: “Marker-trait associations (Supplementary Table 5) were considered significative using a Bonferroni-corrected significant threshold of α∗=α/m with α=0.5(−log10(p)>5.28).

Also, in the ‘Statistical analysis’ section, the first sentence has been corrected to read:

“Adjusted phenotypic mean of each genotype across years (2017–2021) was estimated using a linear model with genotype and year as fixed effects (Jung et al. 2020) as the following:”

These changes have been made to the published paper.

