# Supplementary material for: A bittersweet symphony: genetic insights into cider apple fruit quality
Source: G3 (Bethesda). 2025 Oct 19;16(1):jkaf241. doi: 10.1093/g3journal/jkaf241 (PMC12774593; doi:10.1093/g3journal/jkaf241)
Supplement: jkaf241_Supplementary_Data [file jkaf241_supplementary_data.zip › Supplemental_Figure_Legends_G3-2025-406078.docx]

**Supplemental Figure Legends**

**Figure S1.** Pairwise scatterplots and Pearson correlation coefficients between titratable acidity (TA), pH, soluble solids content (SSC), total phenolics (TP), glucose, fructose, sucrose, and sorbitol. The significance levels of correlations are indicated as follows: *P < 0.05, **P < 0.01, ***P < 0.001. The red lines represent linear regression fits for visualizing trends.

**Figure S2.** Principal Component Analysis (PCA) biplots illustrating the relationships among fruit quality traits across four dimensions (Dim 1–Dim 4). Each subplot represents a pairwise comparison of dimensions, showing the distribution of samples (black dots) and the contribution of phenotypic traits—titratable acidity (TA), soluble solids content (SSC), pH, total polyphenols (TP), glucose, fructose, sucrose, and sorbitol—to the principal components. The color gradient indicates the cos² values of the traits, reflecting their importance to each dimension. Percent variance explained by each dimension is indicated on the axes.

**Figure S3.** Distribution of adjusted phenotypic means for fruit quality traits in the cider germplasm. Histograms show the distribution of titratable acidity, pH, soluble solids content (SSC), total polyphenols, glucose, fructose, sucrose, and sorbitol. Ranges, means, and standard deviations (SD) are indicated for each parameter.

**Figure S4.** Manhattan plots SSC, Fructose, and Sorbitol content. The x-axis indicates the position of SNPs on each chromosome, and the y-axis represents the -log10 (P-value) for each SNP. The dashed horizontal line represents the Bonferonni-corrected significant threshold. Alternating colors distinguish different chromosomes.

**Figure S5.** Genotypic effects of SNP 7966 (Chr16 - 3.16 Mb) on pH. To assess differences in phenotypic values among genotypes, a Kruskal-Wallis test was conducted. Post-hoc pairwise comparisons were conducted using Dunn’s test, with Holm’s adjustment applied to control for multiple comparisons. Violin plots show the distribution of phenotypic values across the three genotypic, with median values indicated. Pairwise comparisons were conducted using Holm-adjusted P-values.

**Figure S6.** Principal Component Analysis (PCA) of genotyping data (253 genotype and 9,566 SNP markers). The figure displays pairwise scatter plots for the first five principal components (PC1-PC5), with the percentage of variance explained by each component indicated on the diagonal. Each dot represents an individual sample.

**Figure S7.** Heat map of kinship matrix with the tree shown on the left. The heatmap illustrates the pairwise kinship coefficients among accessions, with warmer colors indicating higher relatedness.  Dendrogram on the left shows hierarchical clustering based on the kinship matrix.
