# Supplementary figures and images for: A bittersweet symphony: genetic insights into cider apple fruit quality
Source: G3 (Bethesda). 2025 Oct 19;16(1):jkaf241. doi: 10.1093/g3journal/jkaf241 (PMC12774593; doi:10.1093/g3journal/jkaf241)

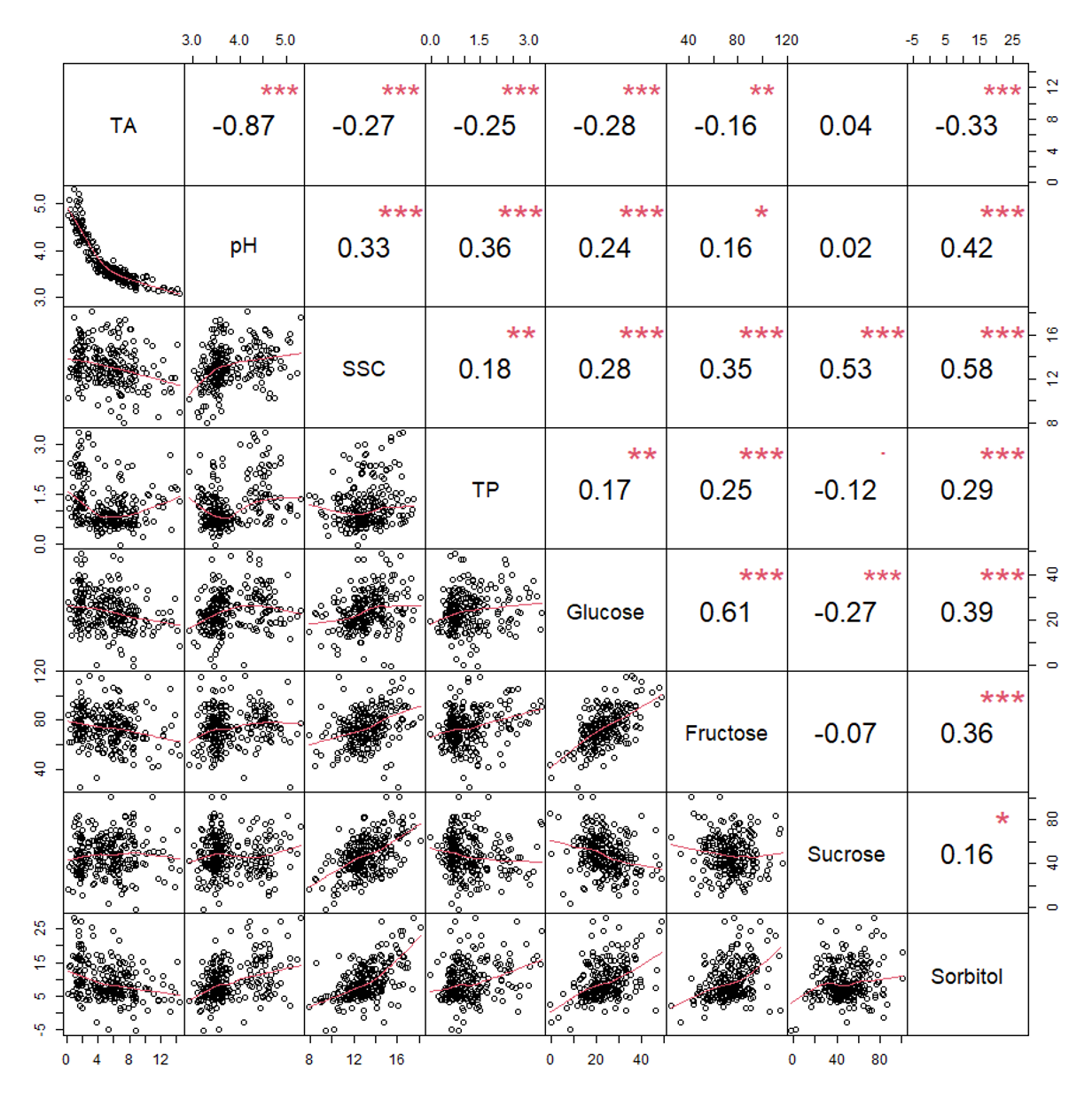

Supplement: jkaf241_Supplementary_Data [file jkaf241_supplementary_data.zip › G3-2025-406078R1_Figure_S1.png]

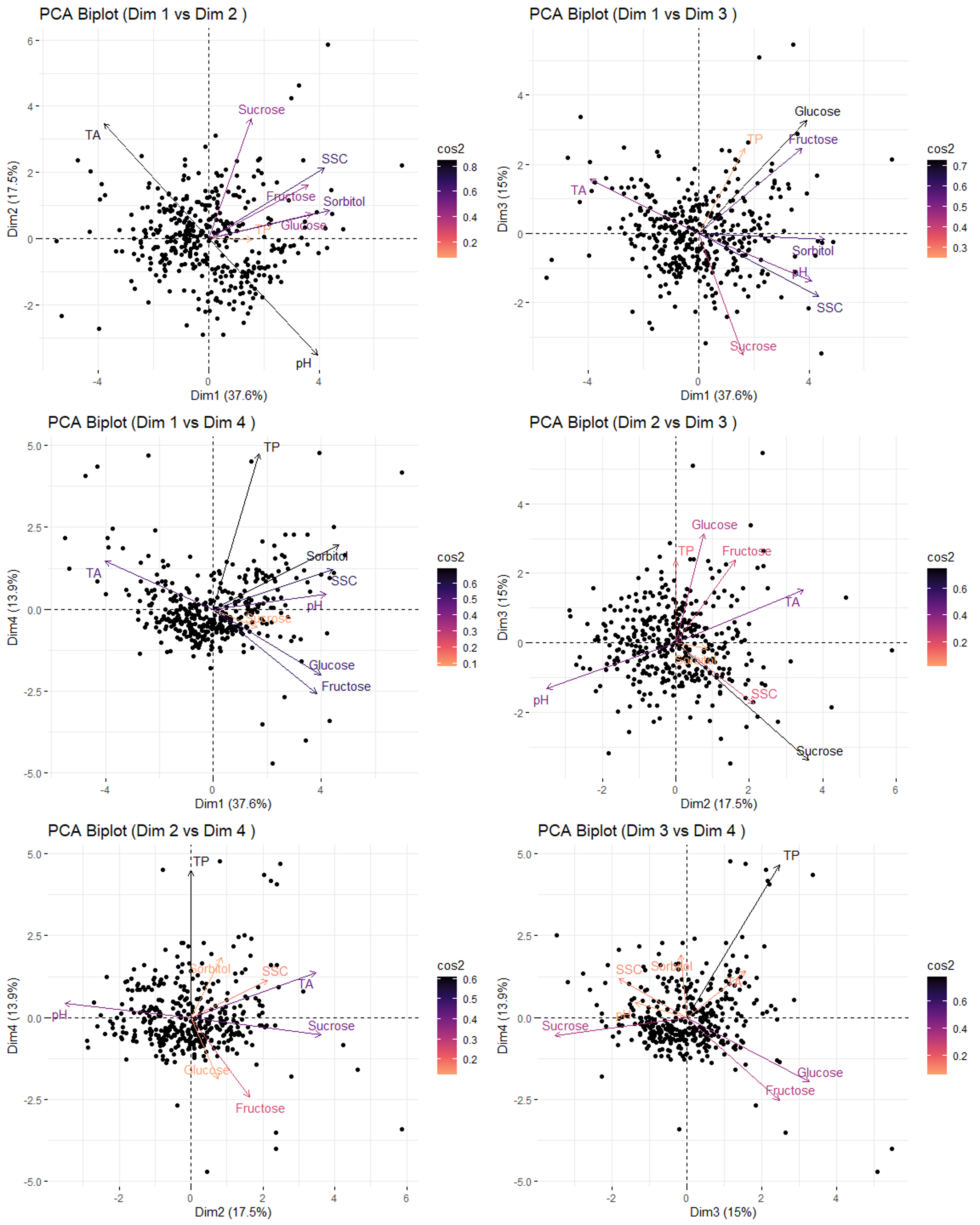

Supplement: jkaf241_Supplementary_Data [file jkaf241_supplementary_data.zip › G3-2025-406078R1_Figure_S2.png]

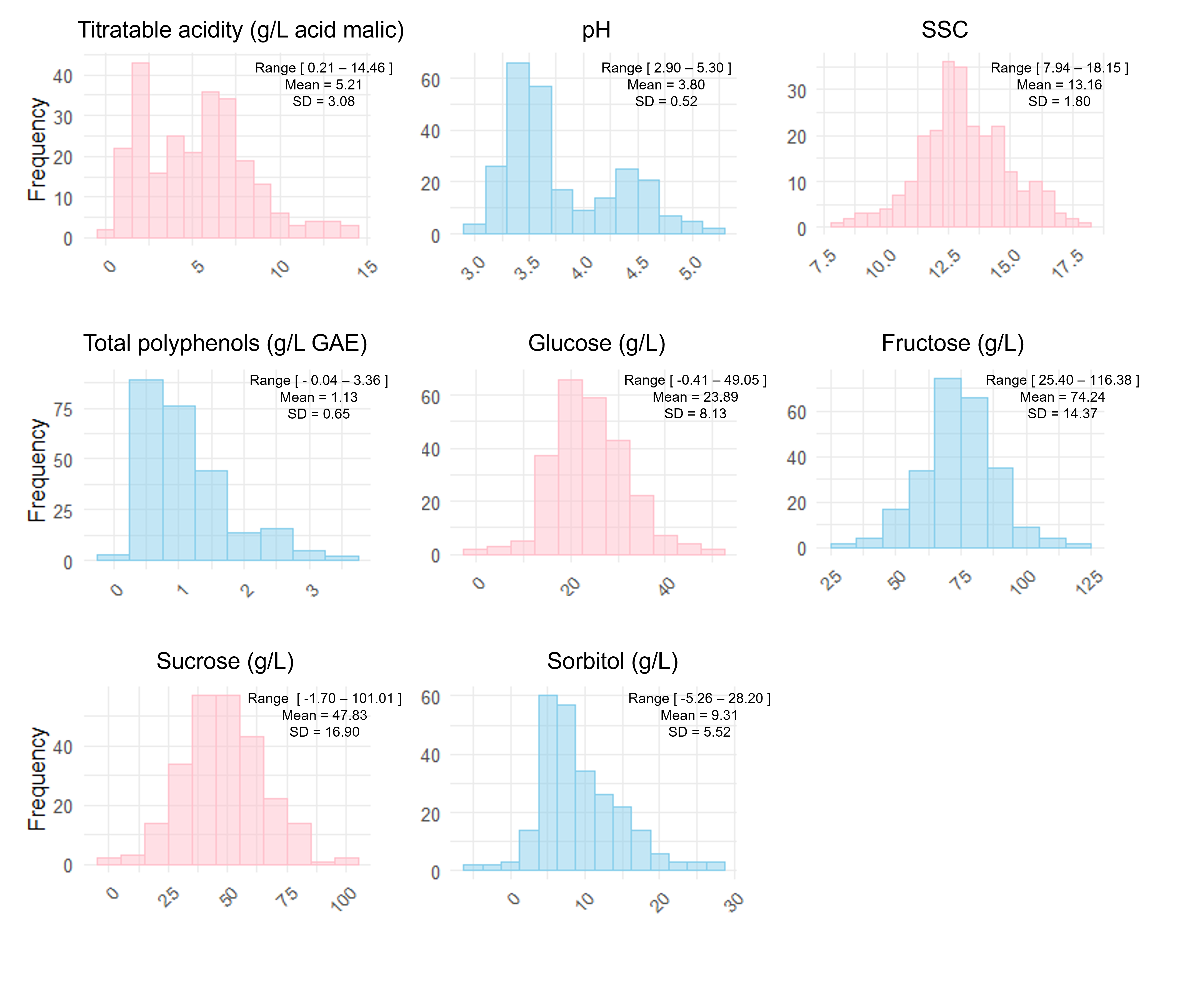

Supplement: jkaf241_Supplementary_Data [file jkaf241_supplementary_data.zip › G3-2025-406078R1_Figure_S3.png]

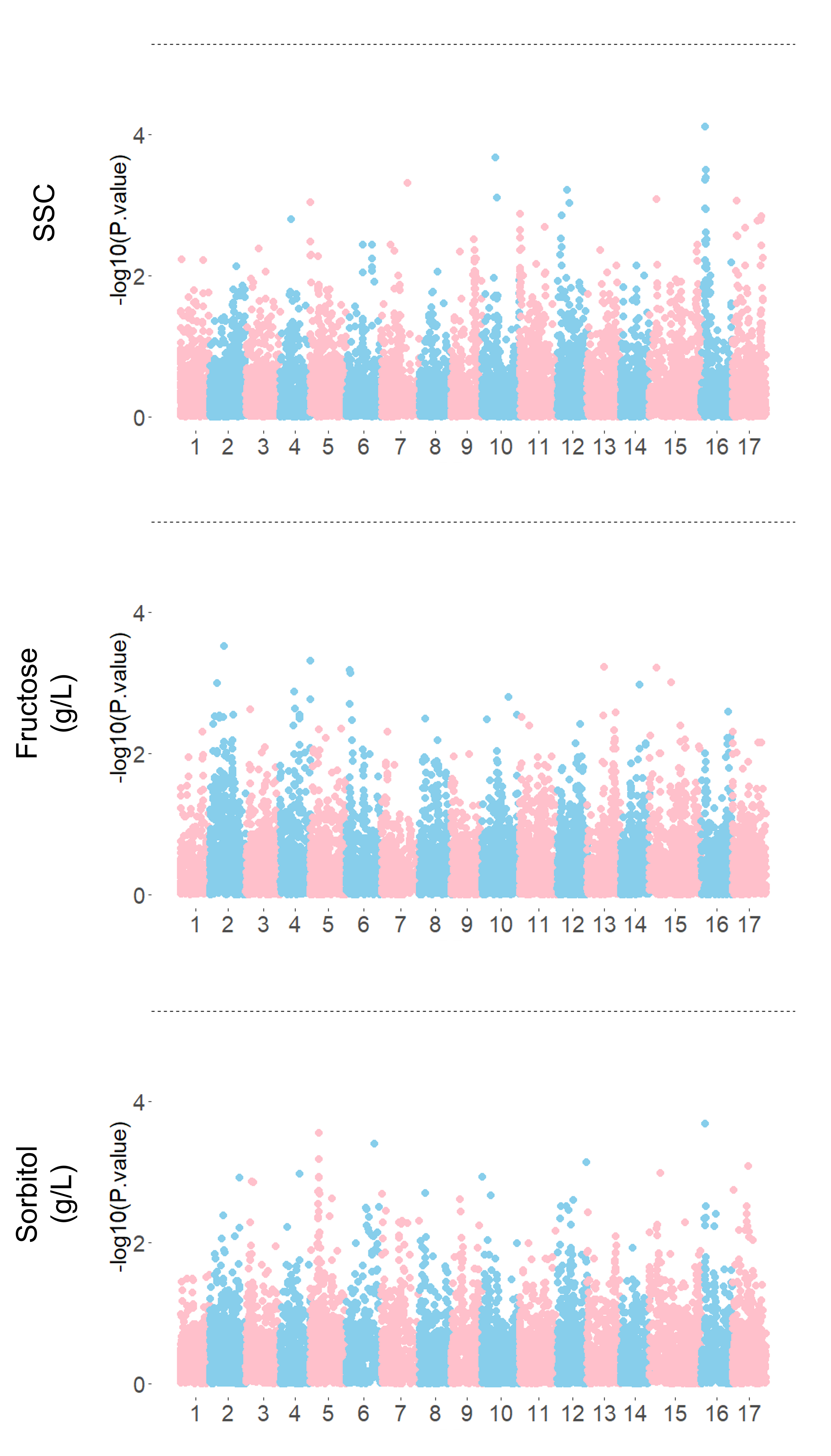

Supplement: jkaf241_Supplementary_Data [file jkaf241_supplementary_data.zip › G3-2025-406078R1_Figure_S4.png]

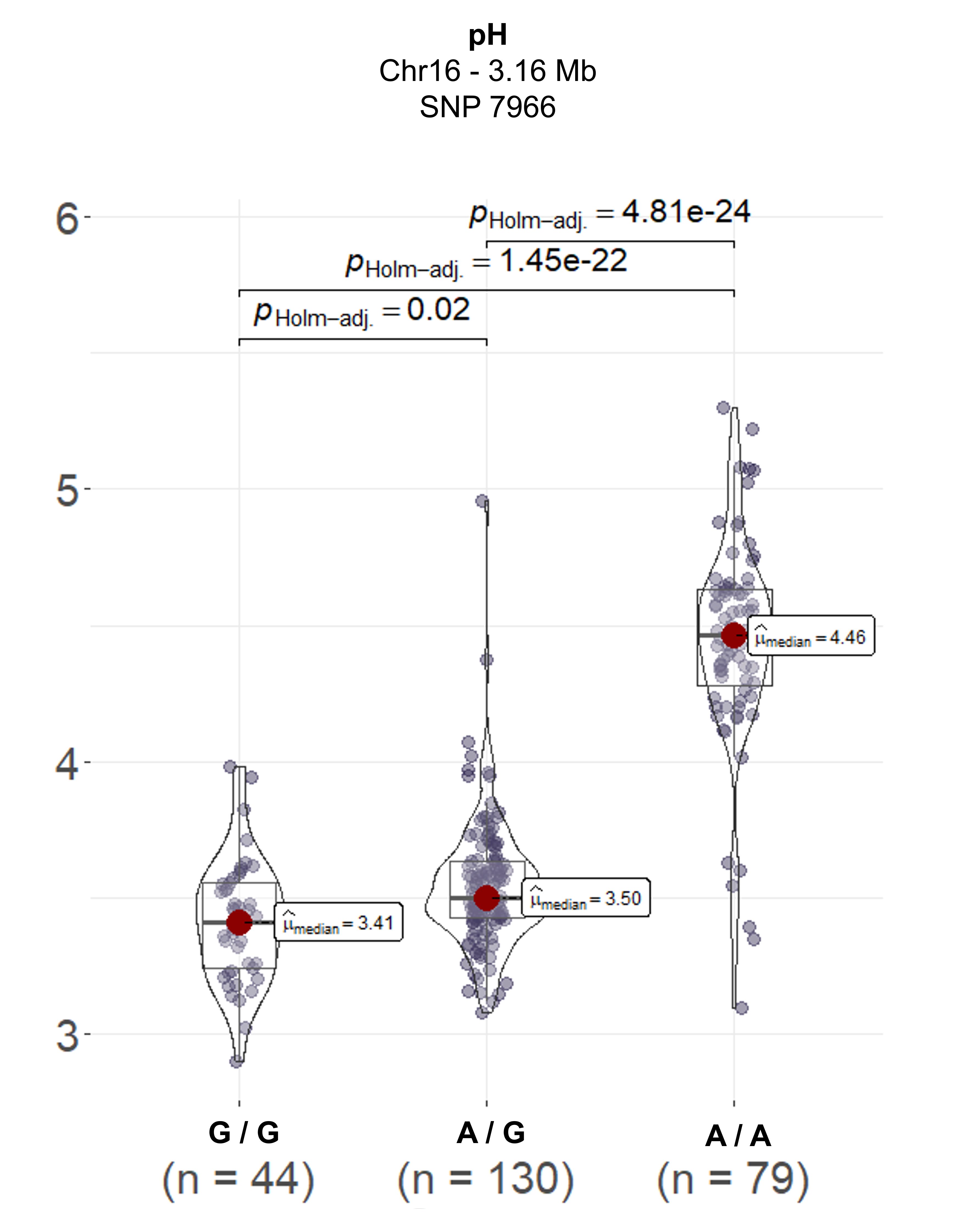

Supplement: jkaf241_Supplementary_Data [file jkaf241_supplementary_data.zip › G3-2025-406078R1_Figure_S5.tif]

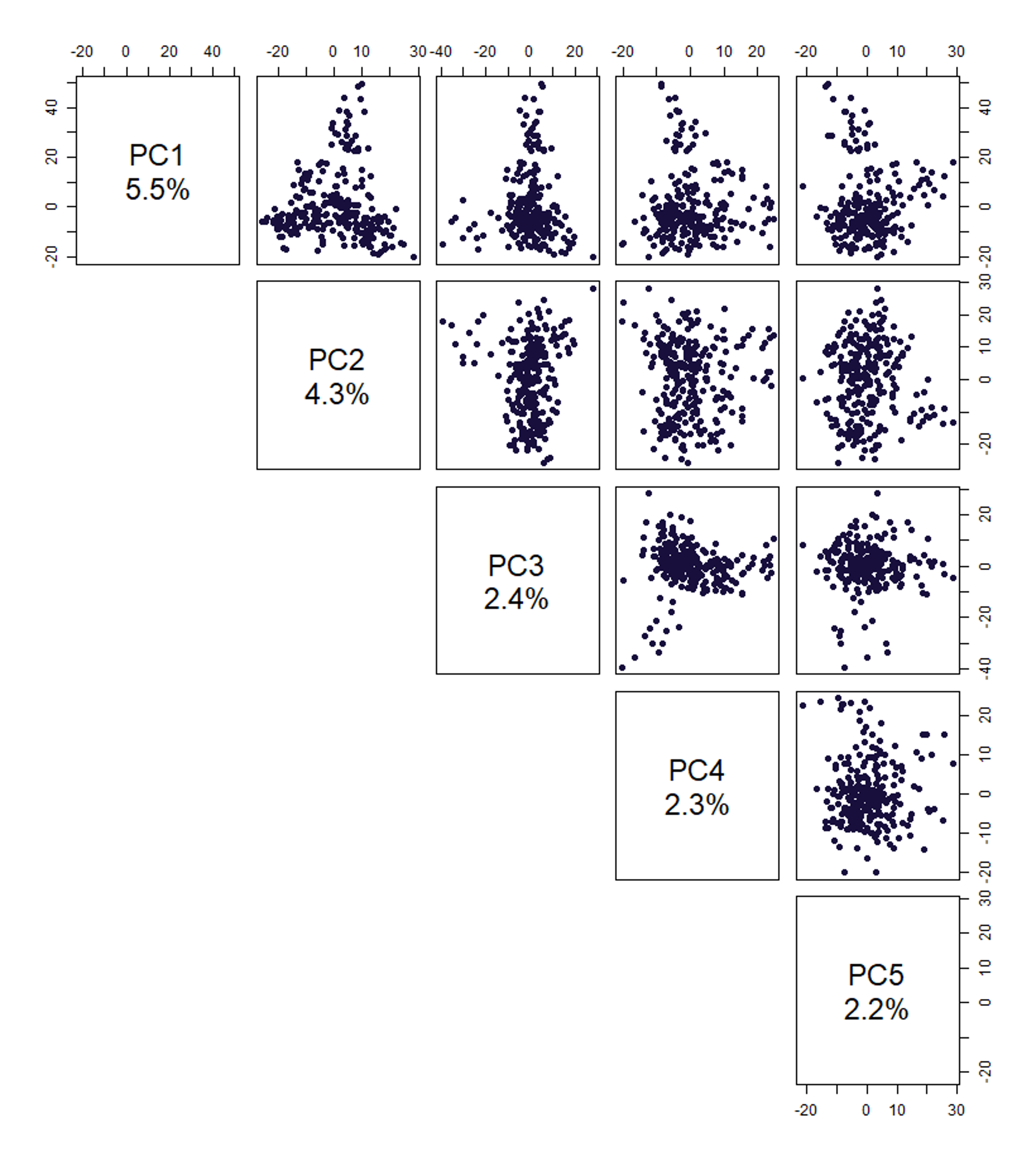

Supplement: jkaf241_Supplementary_Data [file jkaf241_supplementary_data.zip › G3-2025-406078R1_Figure_S6.png]

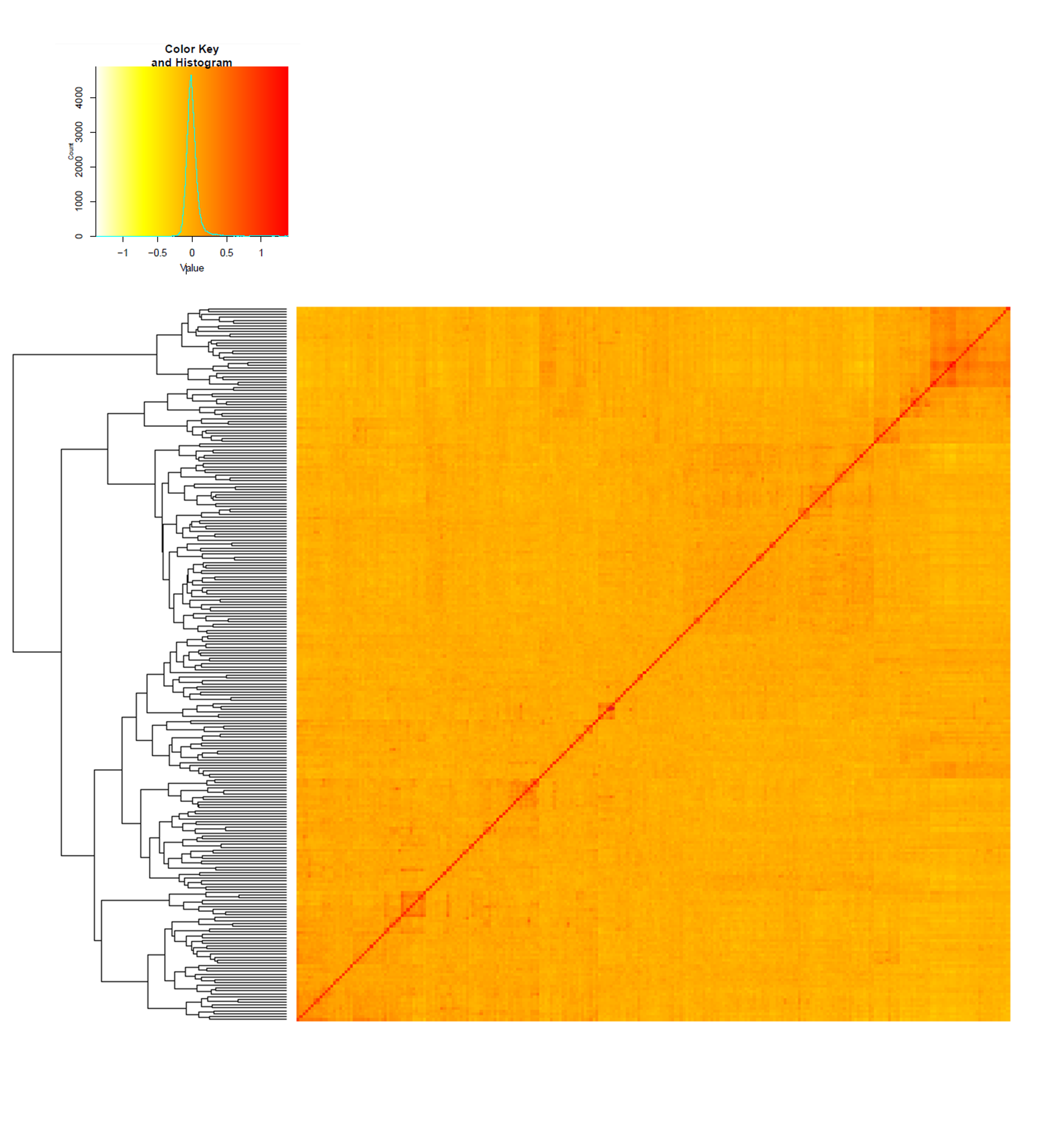

Supplement: jkaf241_Supplementary_Data [file jkaf241_supplementary_data.zip › G3-2025-406078R1_Figure_S7.png]
